# Heart's Ease: Eudaimonia, Musicking in the Pandemic, and Its Implications for Music Education

**DOI:** 10.3389/fpsyg.2021.698941

**Published:** 2021-09-09

**Authors:** June Boyce-Tillman

**Affiliations:** University of Winchester, Winchester, United Kingdom

**Keywords:** eudaimonia, music education, environment, contemplation, relationship

## Abstract

This article will review the themes found in the literature on eudaimonia: ethical behaviour, a sense of meaning and purpose, autonomy – being able to make wise decisions and manage behaviour, contemplation, relationship with spirits of the ancestors and celestial beings and relationships of mutuality, respect. It will use these to critique various events online during the pandemic, such as the Embodiment conference, the SHIFT conference and the ZOOM peace choir. These developments related to music and wellbeing will be used to interrogate the purposes of music education and what might be learned from these new developments in relation to technology in relation to themes, such as values, orality and literacy, process and product.

**The Music of April's Stillness**

There is a stillness deepdown things today

I can nearly hear the worms' journeyings

and the wriggling of the tardigrades in the water butt

The curving edges of the skyclouds are no longer straitened by crosslines

and the fountain burbles an undertone

The falling catkins noiselessly pattern the sunmottled lawn

and the scrabble of the squirrels offers a tooloud rattle

in the pianissimo symphony

The burgeoning green leaves

Will soon be big enough to rustle

Finely tuned branches

perform a graceful dance of varying rhythms

over the bench on which I sit.

All is at rest.

And I long to join^1^.

## Introduction

I start with this poem to indicate how the perception of the sound world has changed during the pandemic, maybe a shift to *Heart's Ease* which is how John Tavener saw the purpose of music (Boyce-Tillman and Forbes, 2020). In earlier work, I charted the development of the idea of eudaimonia from the Greek civilisation in Aristotle through its Christianization at the hands of Thomas Aquinas and how it fed into the positive psychology movement (Boyce-Tillman, 2020). From this review I distilled a number of features within it:

- Ethical behaviour (including towards the environment)
- A sense of meaning and purpose (which may include a cosmic dimension)
- Autonomy (including positive core values)
- Contemplation (which may or may not include a sense of a higher power beyond the world)
- Relationship with spirits of the ancestors and celestial beings
- Relationships of mutuality, respect (including nurture and justice)

I shall use these as analytical lenses to identify trends in the relationship between eudaimonia and music/sound during the pandemic and how these might be reflected in educational practise.

## Musicking, Eudaimonia, and Education

This article brings Small's (1998) concept of musicking together with eudaimonia in the light of internet offerings during the pandemic and the implications for music education. Music education has traditionally concentrated on making and understanding musical sounds which is how David Elliott developed his verb musicing:

The term *music-ing* is a contraction of music making. I shall most often use *musicing* in the collective sense to mean all five forms of music making: performing, improvising, composing, arranging and conducting (Elliott, 2021).

This encompasses a great deal of what goes in in music education; but Small's musicking includes context, values and a variety of interrelationships happening in a musical event. It is concerned not only with musical work (s) which is now seen as a social action:

Music is thus not so much a noun as a verb, “to music”. To music is to take part in any capacity in a musical performance, and the meaning of musicking lies in the relationships that are established between the participants by the performance. Musicking is part of that iconic, gestural process of giving and receiving information about relationships which unites the living world, and it is in fact a ritual by means of which the participants not only learn about, but directly experience, their concepts of how they relate, and how they ought to relate, to other human beings and to the rest of the world (Small, 1998: 9).

In musicking, all relationships – the totality of the experience including listening and setting up the environment are included. The offerings from the internet, which we shall look at initially in their claims for eudaimonia, require us to look beyond the sounds produced by human beings to a much wider sense of a vibrating world to which musickers contribute in a variety of ways:

These ideal relationships are often extremely complex, too complex to be articulated in words, but they are articulated effortlessly by the musical performance, enabling the participants to explore, affirm and celebrate them. Musicking is thus as central in importance to our humanness as is taking part in speech acts, and all normally endowed human beings are born capable of taking part in it, not just of understanding the gestures but of making their own (Small, 1998: 9).

In summary, this taking part includes receiving and contributing in much more varied ways than in the live musicing pre-pandemic. This article asks of music education, whether this expansion needs, or even can, enter the school music curriculum, in order to increase an awareness of the power of music in the pursuit of eudaimonia.

## Cultural Change

There are some who regard the pandemic as a testing time, while others who see it as a time of new beginnings, of redeeming past ills, particularly in the context of the earth, self-reflection and exploration and cosmic eudaimonia. The ready availability of the internet and the extra time many have available have led to the marketing of a huge range of techniques involving music for survival and transformation. These digital offerings regularly involve a search for the spiritual, often outside of established religions or with practises separated from their religious roots; so spirituality is frequently found in the offerings for well-being in many of the internet courses (Puna and Tiatia-Seath, 2017); but definitions of the term vary.

Changes are appearing in the ways musicians themselves - from both academic and professional backgrounds - approach and understand music. In a conference on embodiment^2^ Koji Matsunobu writes:

With a background as a performing pianist, I will address … how embodiment can be cultivated to explore subjectivities and cultivate care and spaciousness, for self and others … I will explicate a form and process of musical engagement, such as harvesting bamboo, manufacturing an instrument, and making a sound, as leading to an embodied experience of nature and place … I will explore numerous methodologies for cultivating intuition, so that trusting the heart, or listening to the gut is fundamental to sorting through the facts (Matsunobu, 2020)^3^

This latter quotation contains many of the tropes that are found in the current spiritual search – embodiment, intuition, the heart, the natural world, the rejection of conventional religion, and a search for new ways. The developments have included sounds that in previous conventions would not be called music and many others besides. Kahn (2001) in a book entitled provocatively *Noise Water Meat; A History of Sound in the Art* identifies a plethora of sound experiences in contemporary society:

noise, auditive immersion in spatial and psychological domains, inscription and visual sound, the universalism of *all sound* and panaurality, musicalization of sound, phonographic reproduction and imitation, Cagean silence, nondissipative sounds and voices, fluidity at the nexus of performance and objective (Kahn, 2001: 3).

He also includes in his list “actual sonic or auditive events or ideas about sound or listening, sounds actually heard or heard in myth, idea or implication, sounds heard by everyone or imagined by one person alone, or sounds as they fuse with the sensorium as a whole” (Kahn, 2001: 3).

The view of sound as embodied rather than simply being situated in a traffic between ear and brain was influenced by Burroughs (1964). He influenced DeLeuze and Guattari (1987), McClure (1963) (who was concerned with large ecological systems and a spiritual understanding of deep ecology), and Artaud (1976). From them emanated a merger of developments in biology and neuroscience with complementary Eastern and Western body practises, reflected in many of the sound offerings for well-being. Some of the suggestions generated by this merger are simple and can be carried out alone; but others are highly commercialised with varying sums required to buy courses – some of which are sound filled – for achieving happiness. This article will examine what is available, starting with those which can be self-initiated and then moving to the more commercialised industry of wellness.

## Self-Initiated

Many of these musical suggestions for well-being concern relating with nature again in a sound-filled way as seen in the opening poem. Whidden and Shore (2018) identify the way in which human beings initially created music for outdoors that would necessarily include the natural environment, and how with the invention of building houses, they created anthrophonic music for indoors which was independent of other-than-human sounds and indeed attempted to exclude them. After this, music became composed and transmitted electronically, enabling new mergers of the sounds from the human and the other-than-human world.

The cost of the separation between the human and the other-than-human world has generated a longing in contemporary society. It is included by Noddings (2002) in her category “spiritual” and is powerfully related with the “I/Thou” experience described by Buber (1970):

But it can also happen, if will and grace are joined, that as I contemplate the tree I am drawn into a relation, and the tree ceases to be an it (Buber, 1970, p. 57).

This often means the rediscovery of older Wisdoms. Some Westerners are relearning their connexion with the land from indigenous cultures, sometimes involving musicking. It is now much more acceptable, for example, to sing to plants to encourage growth. There is a greater acceptance of vibration being the substance of the planet with the result that in making music we engage with the very stuff of the natural world, as set out by Daniel Barenboim in his Reith lectures (Barenboim, 2009).

Indigenous societies (Ellis, 1985) did not see the animate and inanimate worlds as separate; the entire world was seen to have its own energy or quality:

Did you know that trees talk? Well they do. They talk to each other, and they'll talk to you if you listen … Trouble is, white people don't listen. They never learned to listen to the Indians, so I don't suppose they'll listen to other voices in nature. But I have learned a lot from trees; sometimes about the weather, sometimes about animals, sometimes about the Great Spirit (Tinker, 2004: 105–25).

Macy and Brown (2014) assert that such as a sense of separation is the root cause of ecosystem collapse; the severed relationship between music and the other-than-human world needs re-establishing to heal the traumas that our alienation has caused, which can be based on music as a uniting force for creation as a whole as in mediaeval Europe (Boyce-Tillman, 2000b).

Krause (2013) divides the sounds of the world into geophony (made by the natural world), biophony (made by animals), and anthrophony (made by humans). He sees, in the contemporary world, how anthrophony is drowning out the other two. As the opening poem shows, many people have rediscovered biophony and geophony during the pandemic. Krause describes how early musicking (around 1200 BCE) had an intimate connexion to nature:

Even the musical instruments of early humans – the bone, stone, and reedlike artefacts discovered on forest floors and in caves – had ranges of expressiveness … freeing musicians to expand their creations outside the limits of wild sound (Krause, 2013: 142).

Although our musical instruments still use these natural materials, the relationship between the human and the other-than-human world has been fractured. However, new instruments are emerging in which their origins are clearer such as in pipes made from tree branches. The climate emergency is reflected in soundscapes:

We're also losing a legacy of music, languages, and ways of seeing, knowing, and living … The combination of shrinking habitat and increasing human pandemonium has produced conditions under which the communication channels necessary for creature survival are being completely overloaded. At the same time, we are denying ourselves an experience of the wild natural that is essential to our spiritual and psychological health – a source of rooted wisdom that we simply can't acquire from other aspects of our modern lives (Krause, 2013: 206–7).

Krause likens this drowning out of geophony and biophony to reducing the size of an orchestra (Krause, 2013: 203). He critiques Thomas Aquinas in the history of eudaimonia, for establishing the notion of the soul as unique to the human world and so losing touch with the music of the voices of animals (Krause, 2013: 142). This, claims Krause, was the entrenching of the “view of the natural world as resource.”

With the current development of recording equipment, composers are producing aural symphonies from the natural world. The composer Matthew Herbert^4^ has produced an album called *A week in the life of tree* portraying 180-year-old black pine tree (which includes much birdsong), suggesting that it will be better for people to listen to this rather than politicians' debating. His composition *The State Between Us* (2019) includes sounds of empty harbours, factory demolition and the calls of animals nearing extinction in the interests of tolerance and compassion^5^. Chris Watson in a radio programme entitled *A small slice of tranquillity* examined the nature of acoustic tranquillity and found:

breathing, footsteps, a heartbeat, birdsong, crickets, lapping waves, and flowing streams – researchers demonstrated that such sounds stimulate the limbic system of the brain, resulting in the release of endorphins and a feeling of serenity. Watson eventually concluded that tranquillity refers to a basic layer of sound – an elemental acoustic foundation – upon which we can rest our mental processes (Quoted in Krause, 2013: 216–7).

This has been an important contribution to soundful eudaimonia (Krause, 2013: 206–7). Researchers at Brighton and Sussex Medical School found that:

playing “natural sounds” affected the bodily systems that control the flight-or-fright and rest-digest autonomic nervous systems, with associated effects in the resting activity of the brain … Participants listened to sounds recorded from natural and artificial environments … When listening to natural sounds, the brain connectivity reflected an outward-directed focus of attention; when listening to artificial sounds, the brain connectivity reflected an inward-directed focus of attention, similar to states observed in anxiety, post-traumatic stress disorder, and depression … The study of environmental exposure effects is of growing interest in physical and mental health settings, and greatly influences issues of public health and town planning^6^.

This clearly sees the negative effect on human well-being of the invasion of a usually unacknowledged sound environment by noisy commercial interests such as motorcycles and Jet skis (Krause, 2013: 175), because humans are denied access to the biophonic world (Krause, 2013: 214). In one internet offering, authentic leadership is sought through:

an immersive music experience with her many acoustic instruments from around the world … She weaves those melodies with wisdom traditions, offering a space to step out of your noisy environments and connect with your inner guidance^7^.

Our noisy world has caused an increasing fear of approaching silence which, in situations like an anechoic chamber, humans find extremely difficult. Below, I describe one way to address this in a music classroom. The SoundTracker, Gordon Hempton, writes:

The Hoh Rain Forest at Olympic National Park is the least noise-polluted place in the lower 48 states–an acoustic haven for both park visitors and wildlife that depend on a pristine acoustic environment to communicate. Silence is not the absence of something but the presence of everything^8^

He, like Matthew Herbert, is producing beautiful recordings from environmental sounds which call human beings to leave their own sound-making to attend to the other-than-human world (Hempton and Grossman, 2010).

Composers such as R. Murray Schafer are placing performance in the natural sound world, such as *The Princess of the Stars* (Schafer, 1981), staged at daybreak on a lake. The closure of concert halls and the consequent rise in community projects has placed many more musicians in outdoor spaces. In online courses, people are being advised to play musical instruments in forests:

Make nature “cool” again … [We] recorded folkloric instruments and traditional songs of Elders… [and] was moved by their accounts of deforestation and its impacts on local people^9^.

Composers are celebrating ecological themes, sometimes in the rediscovery of embodiment (Shusterman, 2008). The voice is called the Cathedral of the Self in the vocalisation of this healer:

who sings in ancient mystical resonances, making love audible with her ethereal and earthly voice… clearing out the cobwebs of a vast ancient cathedral within, inviting surrender into power, love and deep peace^10^

So, in a time of change, spirituality, music, silence and the other-than-human world are being brought together in the cause of well-being and changing the very nature of what is considered musicking. For so long, music has been defined as emanating from, and only capable of being created by, human beings; this brief survey has shown that this is being redefined to include a cosmic sonic world. Musicking is no longer simply anthrophony but opens up new ecological possibilities as human beings relinquish the idea of absolute control of the sonic world.

## The Development of The Wellness Industry

This section will chart the line that links the Greek idea of eudaimonia to the current positive psychology movement (Boyce-Tillman, 2020) in areas such as the definition of the self, the pursuit of happiness, morality, societal well-being, the spirituality of contemplation, developments in physics and neuroscience, relationships with the cultural past (the ancestors), and the place of autonomy in a neo-liberal economy. In between Aristotle and the twentieth century came Rousseau's notion of the self. Bentham (1776/1948) developed the notion of the happiness of people as a measure of political success in his doctrine of utilitarianism. Hills's *Think and grow rich* (Hills, 1937) saw a relationship between success and wellness, or more particularly happiness – now regarded as a key to the good life. This was followed by Peale's *The Power of Positive Thinking* (Peale, 1953) and psychologists such as Maslow (1962, 1971) and Rogers (1976).

For Seligman, positive emotion is one of the primary building blocks of wellness (Seligman, 2011), deepening what he saw as the cheap happiness of Vincent Peale's positive thinking from positive psychology, which, in his version, includes developments in neuroscience. Findings in neurophysiology such as mirror neurons (Molnar-Szakacs and Overy, 2006), the release of endorphins (Chanda and Levitin, 2013), and “happy” hormones (Fancourt et al., 2016) effectively draw musicking into the pursuit of well-being. Seligman and Csikszentmihalyi (2000), in establishing the interdisciplinary field of positive psychology, longed for “a common vocabulary of measurable positive traits” (Seligman and Csikszentmihalyi, 2000: 5). William Davies resisted the influence of reductionist psychology (Davies, 2015: 214) because of both its failure to see the human being as a whole and also its desire to control people by means of measurement. Bentall (2004) called for sensitive listening as an alternative to drug treatments; such developments place musicking, with its stress on listening, firmly within well-being strategies.

Gradually the Happiness/Wellness project moved some way from Aristotle's eudaimonia, which was based in virtuous living and action, towards the pursuit of pleasure for its own sake. Eudaimonia is reborn in a form inextricably linked with the development of a market economy. Cederström and Spicer described the emergence of *The Happiness Doctrine* (Cederström and Spicer, 2015: 62–91) which underpins the increasing number of self-help strategies on offer. Neurological issues drawn from Damasio's (1994) critique of Descartes's error saw how the emotional and the rational needed to be brought back together. The individualised heroic journey became a justified choice (Boyce-Tillman, 2000a) with a loss of the more cultural dimensions in the Aristotelian model.

Of the eudaimonic characteristics listed at the outset of this article, justice for societal well-being - associated with political action - is lost in favour of a quite oppressive much more individualised “biomorality” (Cederström and Spicer, 2015: 8), which includes strategies that include finding your inner child, journalling, practising yoga, and hobbies such as bird-watching (Critchley, 2010). A sense of meaning and purpose is encapsulated into life-coaching, which involves keeping track of your own individualised bodily functions and can include various musical practises, such as chanting and drumming. The spiritual vacuum of secularism is generating many mergers of science and various spiritualities:

Neurological, physiological, and behavioural monitoring devices are clamped together with meditation practises and pop-existentialism. The philosophical deficit in the science of happiness is dealt with by importing ideas from Buddhism and new age religions. Somewhere in between quantitative science and spiritualism sits happiness (Davies, 2015: 38).

Contemplation has metamorphosed into mindfulness and combined with “scientific facts about what our brains and minds are “doing” and quasi-Buddhist injunctions to simply sit, be and “notice” events as they flow in and out of consciousness” (Davies, 2015: 259). With no underlying philosophy (unlike the Christianity of Aquinas) but a science-based optimism, happiness can be seen as based in the body or metaphysics. This dilemma provides a space for marketing musicking – with its calming and energising qualities – and its ability to induce a different way of knowing.

Autonomy, in a normalised competitive individualised culture, is how to keep positively motivated and successful. An increase in ill-defined illnesses – a generalised demotivation - that cannot be named as a mental illness was producing increased workplace absence even before the pandemic:

Someone with low symptoms of mental illness but with a negative balance of positive vs. negative emotionality would be in a state of incomplete mental health (Cabanas and Illouz, 2019: 152).

Although these symptoms may well be the result of social, political or economic disempowerment, the treatment of the mind/emotions deflect the causes from organisational systems onto an individualised guilt; this can reduce human ability to make well-considered decisions. People look inwards for solutions (Davies, 2015: 251). “For them [managers] mindfulness becomes a way of shifting the responsibility for social ideals back onto the individual” (Cederström and Spicer, 2015: 25). The denial or pathologizing of negative feelings effectively prevents personal unrest from crystallising into social protest.

The pursuit of happiness cannot trump reality and the pursuit of knowledge - critical thinking about ourselves and the surrounding world (Cabanas and Illouz, 2019: 183).

In this dilemma, the marketing of musical techniques to keep up the necessary energy levels for demanding work contexts fits neatly. Autonomy becomes a process of surviving unjust working conditions, rather than protesting against inhumane practises.

In summary, the development of the wellness industry has moved away of Aristotelian model of eudaimonia by placing it in a neo-liberal context. This can be critiqued for its individualised nature where autonomy and contemplation are valued above ethical behaviour and relationships of mutuality and respect; a sense of meaning and purpose is linked with neo-liberal values. Through this adaptation, success can be more easily measured in terms of increased earning capacity, which itself leads to increased marketability for courses. This industry pulls together the past and the present, merging ancestral practises such as shamanism - perceived through a modern lens - with fragmented selves, digitised technologies and a search for a marketable transcendence. There is a marked shift in the marketing of musicking as process rather than product. There is no professional cheque on the efficacy of marketed musicking techniques or practitioners, which rely rather on comments on twitter feeds and Facebook.

## The Wellness Industry Online

This section will examine varied examples of the digital offerings which include musicking for eudaimonia, to illustrate some of the trends listed in the previous section. The examples used in this article are selected from a variety of contexts including:

- The Embodiment Conference^11^
- The Shift Network^12^

Practitioners – driven by these shifts in musical value systems- are abandoning commercial careers in traditionally shaped musical performance in the interests of marketing musical well-being strategies. For example, the Brothers Koren^13^, who have performed with groups such as Coldplay are now “on a mission to hear the World's song, one Big Voice at a time” by expanding space for creative expression. Purchasers are offered the chance to find their big voice (linked with autonomy) which has been silenced; this includes breathing techniques which figure prominently in many well-being courses. Soma Breath's *Awakening Experience* is led by a person, who has abandoned a career as a pharmacist to lead “a sequence of therapeutic breathwork techniques combined with brainwave music and guided meditation” designed to awaken clients' full potential^14^ (a contemporary version of contemplation). In *Inner Resonant Exploration: An immersive sound experience to connect to your inner leader*, another practitioner changes career from teaching, music theatre, composing and therapy to work in the area of well-being as a sound alchemist. Here the practitioner has rediscovered other value systems in musicking - by an integration of ancient technology and Wisdom schools into contemporary life in the areas of ancestral healing, meaning and purpose and contemplation, which she sees:

As meaningful entertainment, as a tool for healing, as a boost for intuition, as a facilitator for creativity, as a way to harmonise a conversation, as guidance for group retreats, as medicine for inner transformation, as a medium to connect to the intelligence of the body^15^

The *Jazz Leadership* project^16^ rediscovers the well-being aspects of jazz - in relationship to individuals and organisations - which highlights music's capacity to improvise communication and enable people to handle difficult circumstances. Central to their well-being (encouraging mutuality and purpose) practise is listening – active, empathetic, and generative – which will encourage growth, inclusivity, flexibility, communal sensibility, collective purpose, and trust.

Aesthetic value systems are now given spiritual overtones that would have been found in the philosophies of the Middle Ages in Europe (Boyce-Tillman, 2000a,b). Dr Jeralyn Glass^17^ combines quartz crystal bowls with elements of spirituality: her special crystal alchemy invocation is designed to birth new ideas and projects (sometimes in sync with the phases of the moon).

Emotional healing features in many of the well-beings on offer. Forrest Yoga^18^ “incorporates ceremony, music, dance, ancient healing techniques, Veganism, and First Nation Philosophies” to heal emotional trauma in a ceremony using breath, Asana, and music and relations with the ancestors. Others concentrate on particular aspects of emotional well-being such as *Shame and the Voice*^19^ which uses vocalisation to enhance physical, mental, and emotional well-being and so improve autonomy.

Having critiqued the contemporary developments in positive psychology and the wellness industry with some illustrations, this article will examine how the range of musicking strategies on offer fit with the five elements of eudaimonia set out at the start of the article.

## Ethical Behaviour

Ancient wisdoms and the concept of a sacred vibrating universe (Barenboim, 2009) are revisited in the idea of attunement as a form of ethical relationship with the environment. This rediscovers vibration as the essential stuff of the universe, with molecules and atoms circulating in apparently static matter, and vibrations of liquid crystal giving the colour to digital displays.

A renewed interest in paganism and pre-Christian history has led to a renewed interest in sacred sites involving sound such as the *Clach a' Choire* (Ringing Stone) on Tiree's coast; this is an Ice Age boulder which produces a metallic clang when struck - a megalithic portal to another world (Sharkey, 1975). Current veneration takes the form of coins left in a little hollow. Ringing stones in Europe, in Central, Eastern, Southern-Eastern and Western Asia, Africa, South America and are being rediscovered as portals to another world. Music is seen as a profound connexion with the other-than-human world (including the spirit-world and the ancestors):

To the Western mind music is essentially something created by man [sic], although it may be an unconscious process. For the shaman, music is something separate, a form of spiritual power that has an autonomous being apart from human minds (Frowen-Williams, 1997).

These ideas join contemporary science in rediscovering the Earth's hum – “a relentless hum of countless notes completely imperceptible to the human ear, like a giant, exceptionally quiet symphony” (Boyce-Tillman, 2010: 158). This represents a rediscovery of sound as a source for building and healing^20^ Earlier we saw claims that quartz crystal bowls use this energy. New ways of ethical behaviour with the other-than-human world are emerging; there is generalised interest in love but in an individualised world, this is less often linked with virtuous action.

## A Sense of Meaning and Purpose

The relationship with the coaching industry makes this a part of many offerings. A combination of musicking and neuropsychology underpins offerings such as using improvisation to set new goals in a client's life. Sometimes this is linked with sacred wisdom and the use of “exotic” instruments as in *Dancemeditation*^21^ which uses “the hauntingly beautiful music of master oud player and singer, Joe Zeytoonian” to relax clients into an inward search. Titles reflect searching within spiritual practises such as *SOMA – Meditating with The Radiance Sutras*. Here, 50 years of scientific research and practise is combined with “112 doorways into meditative experience through everyday life experience: breathing, sensing energy flows in the body, listening to music, dancing, eating, partying, making love, communing with nature, merging with mantras - even mind wandering and being lonely^22^.” Meaning and purpose are now enhanced by combining science within spiritual practise.

As part of the *Telling Encounters* conference at St Martin in the Fields in London, UK, a group of us associated with the University of Winchester, initiated a music workshop for people with disabilities. Conducted on the ZOOM platform, the workshop was based on poems and pictures from the pandemic around which people improvised and recorded. We also used the traditional song *Scarborough Fair* with its repetition of healing herbs. The image of a finding a way through the woods seemed appropriate for people trying to survive the pandemic and we chose a rondo structure with the university folk group's performance of the folksong as a recurring refrain. Neil Valentine, director of the Winchester University Music Centre, wove the sounds together with sounds and images of the poets; visual images were added from the New Forest by the Rev Rachel Noel. This produced a moving piece – *A Path through the woods -* that gave some sense of movement through and meaning for the pandemic^23^ Here, by using multiple art forms and technology in an innovative way, meaning and purpose could be enhanced.

## Autonomy

The notion of a sense of purpose is often allied with the ability to make wise choices and achieve transformation as shown in titles, such as *Giving Voice to The Body's Tales: How the Voice Can Help Express, Shape, and Transform Our Wounds*. Voice Movement Therapy^24^ aims to free the grip of old storeys that have been hidden, frozen or silenced to enable freedom of choice. *Danse Macabre* claims to heal past traumas through music and silence – sitting, walking, dancing and returning to sitting to experience the movement in our stillness, and the stillness in our movement, based on the work of Gabrielle Roth^25^ Drumming and mindfulness, as in Shusterman's somaesthetics (Shusterman, 2008), appear in *Rhythm Bliss*, which offers a mindful hand drumming, meditation, and movement course^26^

Other less commercial offerings encourage contributions from individuals as well as communal singing with most participants on mute. In Dave Camlin's folk music gatherings, entitled *The Folk Neet*, people are encouraged to contribute songs, poems and films which are affirmed and encouraged – the folk club transferred on line^27^ These can effectively reverse the process of demusicalization by allowing people private exploration (Morgan and Boyce-Tillman, 2016: 53).

## Contemplation

Aquinas's Christian emphasis has given way to mindfulness, drawing on Buddhist practises. *Music and Harmony's* Music and Mandalas project^28^ is designed for elders in care homes; it uses improvised musicking by Alistair Clarkson and Meta Killick – both music therapists - who improvise on harp and guitar using repetitive motifs with calming expressive character - intuitive, sourcing energy^29^. This is combined with colouring mandalas to take participants – both staff and patients - into a liminal space. A powerful mood of mindfulness is created by music which uses repeated motifs in a circular musical shape - a musical cradle, holding the meditative mood. Done in a group characterised by non-judgmental acceptance, the associated tranquillity produces a profound sense of togetherness of patients and staff in a care home context.

There is a vast array of music allied with a variety of meditation techniques, produced by such notions as opening the chakras (Boyce-Tillman, 2000a). *Open Floor Dance* is described as mindful movement “where music meets the internal landscape”^30^ In the *Beyond the Veil Summit* series, Jeralyn Glass illuminates this with conversations with medical workers, researchers, authors, who have had near-death experiences, linking these with her experience of the death of her own son. Seldom are Christian beliefs found in the range of belief systems which are combined with musicking in this field; many offerings have a sense of a world beyond the material one and embrace gods, goddesses, angels and fairies - figures which Enlightenment rationalism and scientific evidence-testing reduced to the realm of superstition.

## Relationship With The Spirits of The Ancestors

Often linked with a spiritual view of re-incarnation, this aspect, which had declined after the Enlightenment, is re-emerging strongly. Jeralyn Glass declares that “Waiting on the Other Side is Unconditional Love.” She links her discovery of these healing powers with the death of her son and her way of dealing with grief – combining musical materials with sacred ideas. With her pure quartz Alchemy Crystal Singing Bowls her teaching includes “variations of alchemies, the size of the bowls, single notes, bowl tunings, and some of the structure and science behind healing sound,” which will enhance the immune and the endocrine system affecting a person's autonomy and their relationship with the spirits of the dead:

With proper guidance, you can integrate insights from those who've “crossed over” and mediated messages of healing and closure.

In *Open Floor Movement*:

As the beat slows down, we feel our lineage, culture and history, and allow waves of life-force energy to connect to our ancestors, bringing healing^31^.

One workshop, entitled *Inviting in the Ancestors: Accepting the Good that Awaits Us*, is run by a Dharma nun in Thich Nhat Hanh's Order of Interbeing; the workshop weaves “meditation, movement, music, mindful breathing, interactive exercises, and reflection to explore the presence of our ancestors in us and how we can include them more in our daily lives to support healing and access wisdom and guidance.” In the *Beyond the Veil* conference the scientific study of consciousness is invoked to make claims that guides, angels and loved ones are ready and eager to help us and that mediumship opens up the higher self and creative energy. The importation of wisdoms from a variety of spiritual traditions has enabled a powerful rediscovery of this area.

## Relationships With Spirits/Daimons

Often ignored on current writing on spirituality, these are part of many of the great faiths including Islam, Christianity and Hinduism. Across the history of eudaimonia, the nature of the daimon has been understood and/or misunderstood in a variety of ways, all playing out in any given time. These understandings range along a continuum which includes a daimon being the essence of the self to being a primal impersonal force originating from a world outside the self. Its evolution into the word demon in Christian theology, European mythology and folklore aligned it with malign power, such as (currently) a force that leads a person into addiction; however, in ancient Greek understanding, there were eudaimons and cacodaimons – good and bad. Theologians have sometimes equated daimons with the Holy Spirit or guardian angels. Sometimes they are associated with a person who has been an important influence on a person's life who becomes a benevolent daimon after death. Socrates saw his daimon as warning him of danger and so preventing bad choices. Indeed, sometimes a person is seen as having two daimons – one personifying the conscience and the other evil desires; this dualism resulted in daimons such as Hecate haunting crossroads in our lives (Roberts, 2019). Sometimes they appeared in animal form or a human and animal mix that used music, such as Pan with horns and goat's feet, who played his syrinx to encourage fertile pastures. Beautiful women nymphs were seen as benevolent beings living in springs and caves, dancing, and singing. These developed into such creatures as pixies, fairies, mermaids, and trolls.

Daimons have been brought into contemporary thought through Pullman's (1995, 1997, 2000) trilogy *His Dark Materials* where they are spelled daemon (Merton, 2003). Pullman's books have the potential to introduce such a discussion into a classroom (Romano, 2001). Many articles have been written about Pullman's concept which liken it to a manifestation of a person's inner self. Pullman (Bobby, 2004) claims that the association of Lyra with her daemon was an important trigger in the writing of the trilogy. In Lyra's world in the trilogy, every human being has a daemon companion, which takes the form of an animal which is as important as the human being. Controlling authorities have attempted in the narrative to separate the human and dæmon in a way which did not result in death but in the loss of willpower and vitality. In childhood, daemons shapeshift at will and take the form of any animal – both attractive and unattractive. It is perhaps the paradoxical nature of the daimon that makes Pullman's world attractive to so many readers. Both ancient worldviews of spirits and Pullman's reworking of them feature in contemporary offerings of musicking for well-being.

## Relationships of Mutuality and Respect

Relationship is often expressed as entrainment when two independent phenomena develop a shared pulse. This leads to special claims for the connective power of music - sympathy and empathy - as “love-as-action” (Silverman, 2012) and that interchanges through musicking (Small, 1998) differ in quality from everyday encounters because they are freely chosen rather than enforced (Bourriaud, 1998: 16). Sometimes empathy with the marginalised is linked justice; so the voice coach *in Embodying Community through Virtual Arts Events* “utilises her art to create collaborative spaces, engage the imagination in all people, and support movements towards justice^32^”

Group activities especially singing are seen as having health benefits (Morrison and Clift, 2012; Clift et al., 2013; NICE, 2015). The musicking space is sometimes described as spiritual or liminal (Clarke, 2005; Boyce-Tillman, 2016a) - a place where individual identities are dissolved (Pavlicevic, 2013: 197); this has helped a new turn towards a community aesthetic (Jackson, 2011: 212) or Bourriaud's “relational aesthetics” (Bourriaud, 1998: 15). As social prescribing (Walker and Boyce-Tillman, 2002) develops in the UK, musicking can provide wholeness in a fragmented society (Storr, 1993; Hinchliffe et al., 2018). It can rebalance a highly individualised health system and contribute to the reestablishment of a nurturing community (Crawford et al., 2013: 137–152; Morgan and Boyce-Tillman, 2016; Camlin et al., 2020). Group musicking has become very difficult in the pandemic; people have felt deeply impoverished by the loss of the sacred initiated by singing together. Virtual choirs are emerging but each voice is recorded individually.

The individualism of the digital process of the virtual choir powered the creation of the ZOOM peace choir at Winchester University, which can also be related to relationships of mutuality and respect (Illman, 2012). Faced with the complex time of COVID awareness, with people unable to congregate, I examined how relationships of mutuality and respect might be established by simultaneous musicking technologically. The inability of the ZOOM platform to support a single shared pulse led to using a drone as a unifying element. I had composed a set of chants based on the same chord, combined with the notion of chance/choice, as part of an event entitled Space for Peace (Boyce-Tillman, 2012, 2013), performed for over 9 years in Winchester Cathedral and Winchester University and also in other faith venues including a Hindu temple in Southampton and St John's church in Hackney. Each event had participants from different faith and spiritual traditions with differing responses and outcomes. These participants included a rabbi singing Jewish cantillation, school choirs, community groups, university choirs of different kinds and the Islamic call to prayer. It saw performance as process rather than product - creating beautiful harmonies by chance/choice methods. Virtual Space for Peace is an adaptation of this by Neil Valentine of the University of Winchester Music Centre^33^ My thinking saw a damaged and struggling world needing a protecting veil of love to enfold it.

The behaviour of the technology gives interesting dimensions to who is heard clearly and who is not. The conventional notions of what is good and what is not is challenged and people have to claim their power by accepting their own contribution as valid. It has given people a sense of singing together in an entirely new way with a new awareness of other participants from many cultures. Participants' comments have included:

- You have to abandon everything you have learned
- I became confident in my singing
- Timing does not matter – that is the gift.
- I was not required to produce perfection
- Initially it was very weird. I had to listen to my own voice. I thought I am not going to be able to make this but became more self-confident as it went on and then I did not want it to stop. I did more improvisation. I felt connected with the rest of the world. It was a good experience.

Some people called it a loom on which the world could weave or a cathedral open to the world:

I really enjoyed … [the] presentation of types of selves … through technology … the music was beautiful as … the combined energies were tangible [and the] higher harmonic beautiful - the opportunity for internalisation of a deeper connexion with the natural world … humanity comes to this place of crossroads. with light and thanks (Participant comment 2020).

Appreciation arose from various faiths:

This space allows us to create ecosystems of hearing and being together “en-semble” in togetherness, that shows us how to positively use technology in a “live” way and in a way that brings forth values that are universally applicable in a kaleidoscopic expression of our pluralism. For indeed, Allah reminds us in the Quran that Allah created us into tribes and nations that we may know one another, that we may know our common origin (Participant comment 2020).

Recently (January 2021) we performed the improvisation in association with Levinsky College in Tel-Aviv. Here, with no shared spoken language, Arab, Jewish, and people of a variety traditions in the UK improvised together and the style of the music shifted between the cultures of the three participating groups.

In conclusion, mutuality and respect especially towards the environment characterises much of the field, sometimes combined with an increasing interest in music and justice, which, as we saw in the general description, is sometimes missing from the wellness industry.

## Implications for Music Education

These manifold linkages of musicking with wel-lbeing present challenges for music education. The philosophy of music education stemming from Reimer (1970) defines the “practical, religious, therapeutic, moral, political, and commercial” aspects of music as non-musical; this attitude still persists in some music pedagogy but does not sit well with the musicking for well-being industry. These developments raise profound questions: What is music education for? How does it relate to the world of music outside of the classroom? A music teacher in a girls' school described how many of the 14-year-old girls wanted to take the option of music. Faced with a syllabus based on the European classical music, they quickly gave up; the syllabus bore no relationship to their own involvement and attachment to music. So how far can these developments in the area of music and well-being find a way in the music classroom and should they? How far is music education to initiate young people into a particular tradition and how far is it to enable pupils to navigate the complex landscape described above to enhance their own well-being?

The market based, competitive economy that has invaded education has had little concern for well-being. In Estelle Jorgensen's *Pictures from Music Education* (Jorgensen, 2011), her Factory and Production model describes a utilitarian approach, which could be compared with the use of music for well-being ends described above with the musical product being replaced by the musicking process. The Production approach has been in place for some time in such phenomena as the Associated Board of the Royal Schools of Music graded examinations in the UK; these do not have well-being high on their agenda and indeed, can be seen to work against it, by creating rejects and failures. Jorgensen's Court and Rule picture emphasises the established rules of practise:

A practise driven by certain expectations, which are based on a systematic body of knowledge … The model's exponents honour tradition and exemplar practise, help to keep alive musical knowledge and wisdom from the past, and emphasise the intellectual character of musical knowing at a time in which sensual and affective elements are often the focus of culture (Jorgensen, 2011: 163–4).

There have been various challenges to this from such figures as Schafer (1967, 1975) re-examining the nature of music, Cage and the exploration of silence and noise (Cage, 1980), Tillman (1976), Odam (1995), Dennis (1970) and Paynter and Aston (1970) on improvising/composing, Boyce-Tillman (1996) on world musics, Campbell et al. (2005) on cultural diversity, Swanwick (1968) on popular music and Krause (2013) on environmental connexion. All of these have influenced practise, but examination syllabi often maintain a central core of knowledge based on Western classical music with a few extras added from time to time. The interface between music pedagogy and well-being will be examined under the headings identified above.

## Ethical Behaviour

The idea of virtues has tended to disappear from contemporary music education, even though in the wider society people are often using music in the management of emotional states. Examining musicians individually does not encourage this. Virtues reappeared in a study of spirituality of music education—coming from a former communist state— by Girdzijauskas (2017). Further research in Finland has been done on children's empathy and prosocial behaviour after a 12-h music programme (Kalliopuska and Ruokonen, 1993). The values of co-operation, inclusion and compassion are reappearing the literature (Dowd, 2019; Shaw, 2019) but there is a real gap between these and the competitive exclusivist values of the wider world including the world of the professional classical and popular musician. On the other hand, the rise of community musicking has found innovative ways to pursue more collaborative musical values.

There is increasing writing about ethics and music education (Heuser, 2017) including, in particular, justice. Levinas's notion of heteronomy says that actions need to take other people into account (Levinas, 1969: 303). We have seen this in courses using jazz and improvisational techniques earlier and essential to them is the concept of listening at the deepest level. Cobussen and Nielsen (2012) show that music is a space where ethics happen - “a substantial means of interacting with them, of letting them appear, of making them experienceable, and of transforming them” (Cobussen and Nielsen, 2012: 23). This links with Bourriaud's Relational Aesthetics - being tuned in to other people (including other music makers and the audience) with a deep sense of responsibility to them. In his book *Black Music Matters: Jazz and the Transformation of Music Studies*, Sarath (2018) shows us how jazz embodies the interplay between individual and ensemble. He calls for the centralisation of jazz in music studies in the US in order to ground American musicians in a core facet of their cultural heritage. A spirituality centred in jazz studies and based on the consciousness-based worldview called Integral Theory offers, in his opinion, skills for transcultural dialogue among musicians. Sadly, with adoption of the hierarchical models from Western classical traditions music leaders has often been closer to a leadership model based on a dictatorship rather than mutual entrainment. Woodford (2004) in *Democracy and Music Education*, examines how the democratic values of freedom, creativity, and contribution to society can be embedded in music education; Karin Hendricks sees the rise of compassion in the pandemic as a call for compassionate music teachers “to plant the seeds of compassion for the future.” (Hendricks, 2018: 12). She concludes her book by suggesting that music is primarily about connexion with others (Hendricks, 2018: 159).

There has traditionally been little reference to the environment in the curriculum (except for some moral songs) even though all musical instruments are made from it, as are human bodies. A musical instrument made from parts of the environment provides an intimate relationship with the other-than- human world – perhaps the most intimate other than the process of eating it as food. Kettleborough (2019) offers suggestions for listening to folk material as a way of entering this area. Traditional societies honoured the tree used in the making of the drum; the player would have various ways of keeping a relationship with the wood of the drum (Boyce-Tillman, 2016a). Our industrialised society with its industrial production lines for musical instruments dislocates this connexion, which needs to re-established in our Western fragmented culture.

I have written with the deliberate intention of ecological awareness and respect include *The Great Turning*. This was written for a variety of community groups including schools and orchestra in response to Winchester Cathedral's year long programme on *The Futures of Capitalism*. Based on a book (Reason and Newman, 2013), it emphasises a cosmic togetherness. It included improvised sections and opened with school children tapping stones together to represent the formation of the world from star dust. Other sections honour trees, the earth, ecological projects, the problems of our economy, and so on. During the pandemic we have been preparing for a global performance made of weaving performances from different continents.

The use of natural materials in the classroom, unrefined by manufacture, is growing. In two pieces, *Between* and *The Great Turning* discussed above, I used quantities of stones knocked together - a rediscovery of lithophones. One example of the construction of a contemporary instrument is by Ela Lamblin and Leah Mann. The instrument is created from 100 river rocks suspended by music wire from a wing-shaped sound box and hanging in a steep arch. Investigating sounds from the raw environmental world and refining them could find a place in music education as well as an awareness of the substances from which musical instruments are made and the breath which fires human singing. Pupils could honour the tree which gave its life for the violin every time they open its case.

In summary, the ensemble nature of much musicking can be seen to encourage virtuous and ethical behaviour but individualised examining practises work against this. Increased awareness of geophony and biophony requires a rethinking of what constitutes musicking and the bringing together of the human and the other-than-human sound worlds. Composing and improvising need to include digital ways of integrating anthrophony with those of the other-than-human worlds as well as working outdoors.

## Meaning and Purpose

The arts have always played a significant part in people's sense of personal and social significance, which is often linked with influencing culture in embracing justice-seeking. Giroux (2011) was concerned about the role of education as a site for interrogating and contesting culture, calling the received wisdom into question. He denounced the classism, racism, sexism, portrayal of violence in Western classical music. Campbell et al. (2005) has been very influential in this area, intertwining music education and social understanding. In *Music, Education, and Diversity* (Campbell and Banks, 2018) she addresses the issue of creating global citizens through music education, exploring such issues as how knowledge is constructed, how prejudice can be reduced and equitable pedagogy. *The Oxford Handbook of Social Justice in Music Education* (Benedict et al., 2018), offers 42 chapters addressing the tension between aspirations towards social justice and the privileging of certain traditions through forms of pedagogy which have perpetuated cycles of injustice. Barton (2018) addresses the variety of modes of transmission that music teachers need to address in socially and culturally diverse contexts. The rise of orality in community musicking in the wider society has freed up the therapeutic uses of music but classroom pedagogy often favours musical literacy (Boyce-Tillman, 2016b). The publication (Yob and Jorgensen, 2020), *Humane Music Education for the Common Good* includes scholars and educators from around the world responding to the recent UNESCO report entitled *Rethinking Education: Towards the Common Good*, which calls for a reaffirmation of humane values. It examines how music education might address social justice, inclusion, individual nurturance, and active involvement in the greater public welfare. These texts breathe new life into the aims of the early 19th century music educators who realised that, for the good of society, a concerted and national programme of music education was needed to inspire youngsters to sing and play, extolling music as a means of humanising society and offering artistic expression for all children; this was revisited in Freire's (1972) “conscientization.” Students can be empowered musically to enable their silenced voices to be heard – a theme seen above in many of the well-being projects. I remember a young Aboriginal man singing in a rock group in Darwin, Australia “Don't touch the grog.” The power of music can be used to reinforce any values, as in the Third Reich; all musickers – in whatever role they are – need to look clearly at the underlying value systems of an event, before they are taken up into the liminal experience (Turner, 1969/1974).

However, values are not an overt part of traditional models of music education (Boyce-Tillman, 2007), which seldom include what DeNora (2013) calls music's affordances. The pandemic has caused many traditionally trained classical musicians to work in different ways and find new values in their own musical lives as indicated earlier. Feminist theorists Drinker (1948/1995) and theorists of informal learning practises (Green, 1997, 1988) have drawn attention to the meaning and purpose of music outside of formal education. Informal and orate learning is developing in the growing community choir movement in the UK with its more inclusive and pluralist strategies; but it is difficult to get the associated personal and organisational skills required in this area into musical curricula (Boyce-Tillman, 2018). Mullen (2017) has described how community musicking can re-invigorate musical pedagogy with pupils excluded from mainstream education. The adoption of a new choral aesthetic emphasising community building, diversity, group collaboration, and relationship alongside the classical perspective emphasising performance, perfection, and virtuosity (Pascale, 2005) has enabled many more pupils to find meaning and purpose in musicking.

In summary, many musicians have refined their own sense of purpose in the pandemic to include a greater sense of intention; the move from the concert to more community focused musicking has initiated some pedagogical changes and opened up meaningful musicking to a wider range of people. Music curricula need to respond effectively to needs of community musicians, reconsidering the range of skills included in their programmes (Boyce-Tillman, 2018).

## Autonomy

In Aristotle, this is the ability to make wise choices. The author (Boyce-Tillman, 2016a) examined the potential role of the teacher as psychagogue to lead people out of the underworlds of late capitalism and neo-liberalism that keep people trapped in cultures of consumerism, inequality, addiction and control. Music pedagogy can grasp the totality of music's potential for liberating these subjugated groups and initiate strategies that will give them autonomy - identities of integrity and hope. This blurs the boundaries between music therapy and musicking by seeing the musicking process as potentially transformative (Ferrer et al., 2005; 17).

How far are there opportunities for pupils make choices in music lessons? Improvisatory activities require multiple roles for the teacher - facilitator, coach, trouble-shooter, helper, motivator, and referee; so, in the end, groups of students can work without teacher intervention. I remember arriving late for a composing session with 9 year olds who were all already working at their composition and simply wanted me to validate the decisions they had already made. This was an approach that my research (Tillman, 1987) helped to establish in the classroom. I explored children's capacity to compose/improvise, at a time when this activity was thought to be limited to a few gifted men. It produced busy active engaged classes, committed to active choice-making in music.

Shusterman's (2008) work on somaesthetics, bringing the body into aesthetics encouraged the development of self-knowledge, facilitated through improved awareness of our feelings and our lived experience in the world; somatic efficacy is seen to strengthen the power of the will towards right action (Powell, 2010). This philosophy is similar to many found in the early part of this article on empowerment through music.

Steel (2014) looks for a restoration of a freeing Dionysian spirituality outside of the curriculum as a way of empowering education, as seen in offerings earlier in this article. This he relates to increased self-awareness when music is freed of the controls of a curriculum. Musicians can and have lead their organisations into joyful choruses even in a pandemic. The University of Winchester Music Centre, practising musicking outside of the curriculum and run on the lines of community music outside of the curriculum, can be seen as providing a “chorus school” for the university as a whole, which draws freely on its expertise for a multitude of university celebrations during the pandemic, powered by innovative technological strategies. It may be that musical well-being flourishes better outside of the demands of a fixed curriculum, where it can respond more flexibly to students' needs and explorations.

In summary, awareness of the empowering effects of making choices in musicking can help pupils' autonomy, which may be better enacted outside pre-planned curricula, because of the way European classical music has traditionally been structured. Improvisatory activities of various kinds encourage pupils' collaborative choice-making.

## Contemplation

Aquinas saw this as linked with the Greek idea of wisdom (but, in his age, situated in Christianity) - the capacity to be reflexive. Musicking can initiate spaces for students to engage in experiences that might contribute to a deeper understanding of human existence (Palmer, 2010). Musicking, in particular, is uniquely positioned as a non-discursive and non-literal phenomenon to express meaning in a unique way (Yob, 2011; Boyce-Tillman, 2017), beyond the trends of standardisation, curricular goals, and normative structures are insights, ideas, perspectives, and abilities (Emmanuel, 2011).

The use of religion in the context of non-denominational education has its problems in several cultures; but there is now a range of spiritualities on offer in the wider world uncoupled from religion as we saw above. The ability to transport us to a different consciousness is often what people require of music outside of education. Music may restore the idea of soul which is very much part of the well-being narratives. Plotkin and Berry (2003) sees the soul as associated with a person's place in the cosmos, not an individualised identity; both the human and the other-than-human soul are seen as ensouled, as in indigenous cultures. Tawnya Smith develops this as rooting it in the earth through music:

where the self is seen as a part of the whole, or as occupying a niche in the overall ecosystem or web of life. Such ecocentric conceptions also go beyond Eastern conceptions of the self that are collectivist but still largely anthropocentric. It is difficult to write about an identity construction where one understands “self as earth” because it is so uncommon and foreign an idea in the English language and culture (Smith, 2020: 10).

In a broader view, contemplation may include the contemplation of the nature of music. Following Schafer (1967, 1975), I loved teaching a lesson exploring silence; in this, a class sat in silence for at least half an hour writing down all the sounds they hear, but, more significantly, coming to terms with their attitude to silence which is often one of fear. Their homework was to find silence at home. Lying on their bed covered by a duvet they became aware of the sounds from their bodies. This always led to a wonderful discussion of life and death and attitudes to silence. Lessons of this kind exploring the very nature of life and death link with contemporary discussions of spirituality; they can include the valuing of silence, solitude and serene meditation (Dresser, 1919: 4–12).

In summary, contemplation in the wider world has moved considerably from Aquinas's Christian addition to Aristotelian eudaimonia and now includes ideas drawn from a variety of traditions; a current interest in spiritualities of various kinds and contemporary developments in the philosophy of music have enabled questions about life, time and space to be debated musically. How far these can be included in music curricula will often be context specific and depend on political attitudes to religion/spirituality:


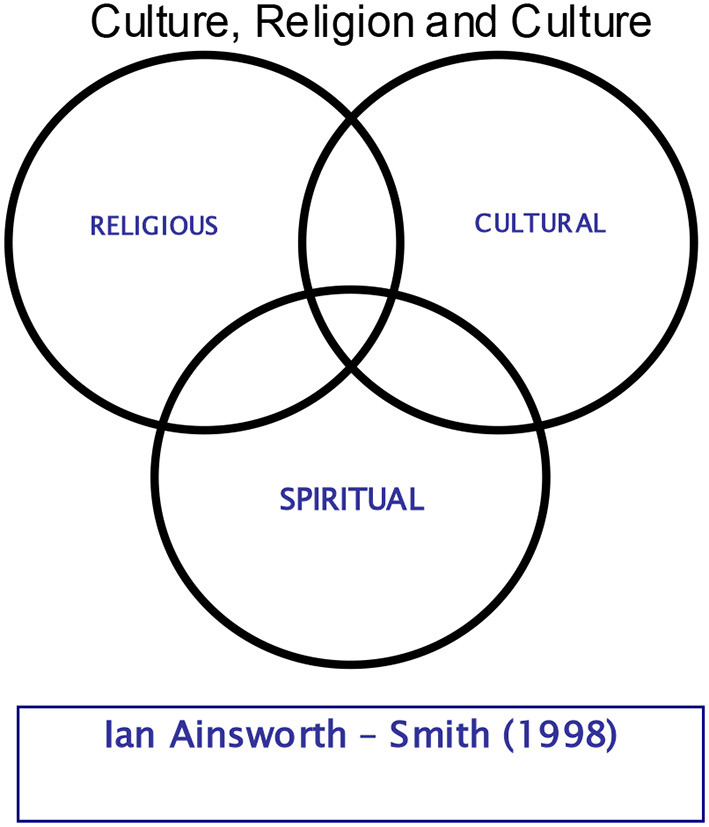


## Relations With The Ancestors

This raises issues of our relationship with the past. The presence of indigenous world views with the presence of ancestral spirits, who can be contacted by musicking - whether it be overtone chanting or a repeated drum beat - offers alternatives to the museum approach to the ancestors which is preferred by Western civilisation. Central to these may be different conceptions of time (Begbie, 2000) and the way that through the medium of music the past impinges on the present. Hans Kung links this with the bliss (or pleasure) of listening to Mozart (Kung, 1992: 27–8).

The music appreciation movement of the twentieth century (Jorgensen, 1987) set up a somewhat contemplative approach to music from the ancestors. In these classes, pupils were asked to listen with wonder to so-called musical masterpieces. Access to the liminal space often happens by recalling (Boyce-Tillman, 1998) and listening to pieces of music from the past. Contact with ancestral spirits in such listening, can be linked with the Greek idea of the daimons and indigenous traditions of spirit possession (Boyce-Tillman, 2008/2009). In the West, there are accounts of musical motifs stimulating a sense of a different self:

I had on Brahms' First Symphony (of which I am especially fond) and was in a state of complete relaxation. However, a chord sounded and at once I was removed from my normal life. My whole physical being dissolved and I knew that I was, in reality, a spiritual creature who only had semblance of a body. I was, quite obviously, the note of music. Not only that, but I was also the light that shone clear blue just to the left of mental vision (Maxwell and Tschudin, 2005: 156).

Such accounts make a case for the continued inclusion of Western classical music in the curriculum, when it might appear to be under threat because of its “elitism, racism, colonialism, and authoritarianism” (Yob, 2020: 125); the Philosophy of Music Education Review (Vol 28 No 2 2020) sets out other ways of approaching it, including “*how* we listen to music is as crucial as *what* we listen to” (Varkøy and Rinholm, 2020: 179). This same article links the vita contemplative to classical music listening through the philosophy of Hannah Arendt (Varkøy and Rinholm, 2020: 178–9). This leads to a discussion of music as art and/or entertainment, especially when we see the word entertainment as coming from the French entretenir, to nurture. If students can be articulate about musical experiences in class, it may enable teachers to help them to evaluate the musical “guides” they are choosing outside the classroom and how helpful or unhelpful they may be; this will enable a further discussion of the cultural construct of musical value. The incorporation in the digital offerings described above of different cultural views of the presence of the ancestors and more celestial beings (see below) represent a rise of more intuitive ways of knowing.

In summary, contacting the ancestors in ways more associated with indigenous cultures could be more freely discussed. This could be interpreted in the light of the classical canon of past composers or discussed more anthropologically in the frames used in other cultures for understanding our relationships with the past. These discussions (including pupils' own experiences and understandings) can inform pupils' ability to understand their relationship with their cultural and personal history and the states of consciousness that music can initiate.

## Relationships With Angels and Daimons

Philip Pullman's use of the daemons opens up the possibility of further discussions of this area in educational contexts - a world that the Enlightenment has deemed mythical. Yet different cultures both geographical and historical have treated them differently and interfaced them with musicking. These accounts - often suppressed in post-Enlightenment Western secular musical culture - are resurfacing in postsecular societies.

David Carr reflects on the use of the word spirit in educational contexts linked with ancient Greece and with clear links to the positive psychology movement:

There is a central and time-honoured sense in which persons of “spirit” are those of strong and positive motivation, and the “dispirited” or spirit-less are those of low energy and weak resolve. From this viewpoint, anything that served to pro-mote greater or livelier pupil application to their studies—such as playing “Rock around the Clock” or “Ride of the Valkyries” during history or physi-cal education lessons—might have some claim to be contributing to their spiritual education (Carr, 2008: 20).

In this area also is a study of angels – celestial beings which were part of European thought until the pursuit of secularisation. Angels were certainly in the spiritual thinking of Thomas Aquinas who, as we saw above, brought contemplation into the concept of eudaimonia. Angels are often portrayed in statuary and pictures as musicking together - linking music with the Music of the Spheres. Classical folk spirituality (Jespers, 2011: 110) includes accounts of angelic presence, as do the experiences of western composers such as Handel in composing *Messiah*:

I did think I did see all Heaven before me, and God Himself seated on his throne, with his company of angels^34^

Musical composition – particularly the generation of musical ideas – has often been viewed through a quasi-mystical lens (Inglis, 2019: 25):

The element of mystery – a sense that something miraculous, beyond rational explanation, is taking place – is a crucial component of the experience of inspiration for most composers (Harvey, 1999: 3).

The incorporation in the digital offerings described above of more celestial beings represent a rise of more intuitive ways of knowing, which were downplayed in the Enlightenment deification of reason and devaluation of intuition. There has been much debate of the relative value of human reason and intuition (Boyce-Tillman, 2005); the interface within human experience of two different but interconnected ways of knowing at some times in our history has been seen impossible and superstitious. How can these two intelligences be held together in educational contexts and can they be linked by musicking?

In summary, Philip Pullman has enabled discussions in this area which could enable some of the musical connexions with celestial beings to be more freely discussed. This could be discussed more anthropologically in the frames used in other cultures for other-than-human-beings and/or accounts from musickers involving beings such as angels.

## Relationships of Mutuality and Respect

Aristotle saw eudaimonia as resulting in relationships that are mutual and respectful. The clearest exponent of this is Noddings (1984, 1992) developed in music education by Dowd (2019) and Shaw (2019). That musical relationships can have a significant effect personally and culturally is being informed by the developing area of community musicking (Boyce-Tillman, 2020) and the development of improvisatory activity.

New issues in this area are raised by technologically mediated music. This has been considerably developed during the pandemic with workshops, improvisation, listening material offered on various digital platforms. Both musicians and pupils have been forced on a steep learning curve digitally with many new programmes and platforms. Traditional listening exercises and their assessment in examinations may well be deemed irrelevant to the ways in which music is heard and created by students outside our classrooms. Whidden and Shore (2018) claim that the different habitats for musicking change the way in which we understand and perceive music. The concentration on the traditional style of classical listening (based on the concert hall) may be ignoring the digital musical environment. To include the understandings of digitally created and received music would restore the inclusion of students' own values to music pedagogy. The classroom would then become a place of discussing people's preferences and of respecting different views and cultures. It would follow Dewey's (1910, 1934) concept of intuition which includes thinking, feeling, perceiving, knowing – emotional intuition – which he saw as encouraged through reflection on action - in this case on well-being in music.

In summary, by starting from the variety of pupils' own experiences of musicking requires a more person centred approach to pedagogy to discover commonalities and differences in experiencing; treated reflectively and non-judgementally such sharings can also generate respect for different ways of understanding and perceiving.

## Summary

This article has reviewed where eudaimonia and music are sitting in the wider world especially in the pandemic and asked various question of music teachers. How far can music education learn from these developments? How far can it help people negotiate a path through them? What is the purpose of music education? Initiation into a particular culture/style? Literacy or orality? Preservation or rebellion? Happiness or eudaimonia? Relationship or autonomy? Hierarchy or democracy? Intellectual challenge or heart's ease? Values or technical skill? Are these mutually exclusive? For a long time, music education has been predicated on the needs of a professional classical musician with a concern for creating perfect products. I took my professorial title, Professor of Applied Music, because I was more interested in what music can do for people rather than what people can do for music. The well-being industry calls education to be concerned with understanding the musicking process and its effect on people's lives – both everyday and spiritual lives. This review offers the possibility of music education - when the totality of the experience is included (Boyce-Tillman, 2016a) - as preparation for the work of living eudaimonically.

## Data Availability Statement

The original contributions presented in the study are included in the article/supplementary material, further inquiries can be directed to the corresponding author.

## Author Contributions

The author confirms being the sole contributor of this work and has approved it for publication.

## Conflict of Interest

The author declares that the research was conducted in the absence of any commercial or financial relationships that could be construed as a potential conflict of interest.

## Publisher's Note

All claims expressed in this article are solely those of the authors and do not necessarily represent those of their affiliated organizations, or those of the publisher, the editors and the reviewers. Any product that may be evaluated in this article, or claim that may be made by its manufacturer, is not guaranteed or endorsed by the publisher.

## Footnotes

^1^By June Boyce-Tillman for the community video project *Unlocking Furzedown* May 3rd, 2020 - NOTE: Tardigrades are very small creatures. They are never more than 1.5 mm long, and can only be seen with a microscope.

^2^https://theembodimentconference.org/.

^3^https://portal.theembodimentconference.org/sessions/panel-embodied-inquiry-3bciaa.

^4^Matthew Herbert Radio4 Today programme UK Dec 17th 7 40 a.m.

^5^http://matthewherbert.com/about-contact/ (accessed December 19, 2020).

^6^https://www.sciencedaily.com/releases/2017/03/170330132354.htm (accessed December 19 2020).

^7^www.laurainserra.com.

^8^https://www.soundtracker.com/products/one-square-inch-silence/.

^9^forestswithoutfrontiers.org; nicodetransilvania.com.

^10^https://www.murielsings.com/.

^11^https://portal.theembodimentconference.org/.

^12^https://theshiftnetwork.com/.

^13^https://brotherskoren.com/home.

^14^https://www.somabreath.com/. This is done by slowing breathing to activate the higher self - generating euphoria.

^15^https://www.laurainserra.com/.

^16^https://www.jazzleadershipproject.com/.

^17^Shift Network – beyond the Veil/ Embodiment.

^18^https://www.ebbflowyoga.co.uk/forrest-yoga/.

^19^https://roisinerskine.com/.

^20^The development of cymatics has produced claims for vibrations to heal and mend organs of the body. http://www.cymatics.co.uk/.

^21^dancemeditation.org.

^22^www.meditationtt.com.

^23^https://www.heartedge.org/main/news/post/316-telling-encounters-a-path-through-the-woods.

^24^https://www.vmtuk.com/.

^25^https://openfloor.org.

^26^http://www.rhythmbliss.com.

^27^https://makemusicday.co.uk/event/merry-neet/.

^28^https://www.youtube.com/channel/UCsgZ6uql3zXNmm92PI66THQ.

^29^Unpublished email from Meta Killick August 18, 2020.

^30^https://www.facebook.com/Anna-Du-V%C3%A9-Dance-and-Shiatsu-249467238923546.

^31^Openfloor.org.

^32^https://www.emilyjeannebrown.com/onvoice.

^33^https://www.facebook.com/WinchesterMusicCentre/videos/564631657821192.

^34^http://supernalliving.com/2012/12/24/legend-of-the-angelic-inspiration-of-handels-messiah/.
